# Real time road scene classification and enhancement for driver assistance under adverse weather

**DOI:** 10.1038/s41598-025-23171-z

**Published:** 2025-11-20

**Authors:** P. P. Anoop, R. Deivanathan

**Affiliations:** School of Mechanical Engineering, Vellore Institute of Technology, Chennai, India

**Keywords:** Machine vision systems, Machine learning, Image enhancement, Convolutional neural networks, Road scenario classification, Computational science, Computer science

## Abstract

Highways are the most widely used mode of transportation worldwide, accounting for the majority of passenger movement. However, the drivers often face difficulties due to poor visibility through the windshield under adverse conditions. In such situations, an alternative mode of vision is essential, and a video display showing the roadway is ideal for this purpose. This paper presents an efficient machine learning-based classification system for various road scenarios, including daytime, nighttime, foggy, and rainy conditions. After classifying the scenario, enhancement techniques are applied to improve the visibility of the road image, ensuring clarity in all atmospheric conditions. Various machine learning algorithms were tested for accuracy in classifying road scenarios, and the most accurate one was selected. Following classification, specific image enhancement techniques were applied to improve the road video according to the identified scenario. A high-intensity mapping technique was used for glare reduction, and a low-light enhancement technique was applied for better night visibility. Defogging and deraining algorithms were employed for foggy and rainy conditions, respectively. An affordable, low-cost system was developed based on the Raspberry Pi 5, utilising a USB camera and a 7-inch display. Compared to state-of-the-art techniques such as Resnet-101 and custom CNN applied for the same kind of work, the proposed model achieves a classification accuracy of 98.67% using the Random Committee algorithm, demonstrating superior performance in roadway classification, even on limited hardware. This approach also shows strong potential for integration into ADAS systems, especially in autonomous vehicles, where larger image datasets and more generalised machine learning or deep learning-based enhancement techniques can be applied. The improved performance of YOLO-based object detection on enhanced images, compared to the original ones, further validates the effectiveness of this method.

## Introduction

**Fig. 1:**
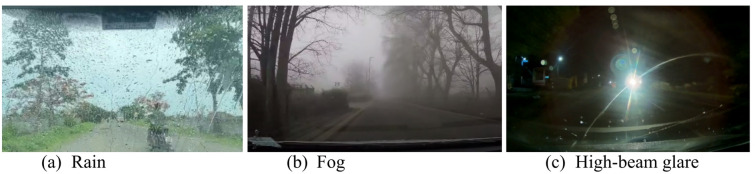
Visual impairments of windshields in various adverse weather conditions.

Each year, more than 1.19 million lives are lost in road accidents worldwide, with an additional 50 million people injured. In this 92% of the world’s fatalities on the roads occur in low- and middle-income countries, even though these countries have around 60% of the world’s vehicles^1^. Poor visibility during nighttime, heavy rain, or snowfall significantly contributes to these incidents, making driving challenging as limited visibility hampers the ability to see the road and spot hazards. Drivers face numerous challenges when dealing with a near-opaque windshield, which can significantly impair their visibility and safety on the road. Figure 1 (a-c) depicts the reduced visibility of windshields in various adverse weather conditions. An obscured view, whether due to fog, rain, dust, or glare, hampers the ability to see other vehicles, pedestrians, road signs, and potential hazards. This can lead to impaired depth perception and delayed reaction times. This reduced visibility increases the likelihood of accidents, such as rear-end collisions and side-swipe accidents, as drivers struggle to judge distances and maintain lane discipline. The constant strain of peering through an obstructed windshield causes significant stress, anxiety, and eye fatigue, further diminishing overall alertness and reaction time. Night driving becomes particularly hazardous, with glare from oncoming headlights exacerbating the opacity issue and temporarily blinding the driver^2,3^. Navigation challenges also arise, with difficulty in spotting lane markings, road hazards, and road curvature, making safe driving tough. These compounded issues underscore the critical need for maintaining a clear windshield and the value of advanced image enhancement and real-time classification systems that adapt to adverse weather and lighting conditions to ensure driver safety and comfort.

Advanced Driver Assistance Systems (ADAS) represent a transformative development in automotive technology, employing a combination of cameras, radar, and LiDAR to continuously monitor the vehicle environment and autonomously regulate various driving functions. These systems provide real-time feedback and automated control to enhance road safety, with features such as automated emergency braking, lane-keeping assistance, and adaptive cruise control^4^. A key component is the front-facing camera, typically mounted on the windshield, which captures live visual data processed by machine learning algorithms to detect, classify, and interpret critical environmental factors. Essential ADAS technologies, including Lane Departure Warning (LDW)^5,6^, Forward Collision Warning (FCW)^7^, pedestrian detection^8^ and Traffic Sign Recognition (TSR)^9,10^, rely on advanced image processing and pattern recognition algorithms to either alert the driver or take preemptive action in hazardous situations, thereby significantly mitigating the risk of accidents. For instance, FCW systems employ shape recognition to assess potential collision threats and engage automatic braking when necessary, while LDW systems continuously monitor lane boundaries to warn drivers of unintended deviations.

Recent advancements in Advanced Driver Assistance Systems (ADAS) focus on the integration of multi-sensor fusion, real-time decision-making, and AI-driven predictive technologies^11,12^. Multi-sensor fusion combines data from LiDAR, radar, and cameras to deliver a comprehensive 360-degree view of the surroundings of the vehicle significantly enhancing object detection accuracy^13^. The fusion of camera and LiDAR technologies mitigates parallax errors and enables reliable real-time decision-making, even under challenging conditions such as fog or rain. Moreover, machine learning algorithms are used to process large volumes of sensor data, enabling predictive responses to rapidly changing traffic situations.

Image enhancement plays a crucial role in ADAS, especially in adverse conditions such as low-light environments, fog, or heavy rain^14^. By employing algorithms that enhance contrast, adjust brightness, reduce noise, and sharpen details, image enhancement improves the quality of the visual data captured by ADAS cameras. This is important for the reliable operation of Lane Departure Warning (LDW), Forward Collision Warning (FCW), and Traffic Sign Recognition (TSR) systems. For instance, during nighttime driving, enhancement algorithms brighten darker areas to show up essential image details, enabling ADAS systems to maintain optimal performance and safeguard driver safety. In foggy conditions, dehazing techniques are applied to clarify images, which is particularly beneficial for functions like Adaptive Cruise Control (ACC), even in reduced visibility.

Beyond ADAS, car manufacturers are exploring additional driver assistance techniques. For instance, anti-glare protective eyewear reduces the blinding effects of high-beam headlights from oncoming traffic, enhancing night driving visibility but potentially darkening low-light areas^15^. Managing high-beam lights is crucial, drivers should switch to low beams when facing oncoming traffic. High-beam assist technology automatically adjusts headlight beams based on surrounding conditions, optimizing visibility without causing discomfort^16,17^. Researchers have explored various methods to reduce glare from oncoming high beams. One method uses Light Dependent Resistors (LDR) and relays to switch from high to low beams automatically when bright headlights are detected^18^. An Arduino Uno-based system alternates beam intensity based on opposing traffic presence and includes a manual switch for control^19^. The Adaptive Front-Lighting System (AFS), introduced by Audi in 2006, integrates sensors, a control module, and actuators to adjust headlight orientation based on vehicle detection^20^. An improved control strategy and kinematic model for AFS allow headlights to adapt both horizontally and vertically according to sensor data^21^. Further advancements include image processing techniques that use binocular cameras to recognize pedestrians, vehicles, and lane markings, adjusting headlight intensities accurately^22^. Another innovative approach uses an LED particle control model, where an onboard camera individually manages each LED in the headlamp system, turning them on or off based on the positions of oncoming vehicles and obstacles. These technologies significantly enhance driver safety and comfort by providing optimal visibility in various driving conditions^23^. High-low beam switching has been a standard feature in modern vehicles for the past few years, helping to improve visibility while minimizing glare for oncoming drivers. However, implementation of the technique across all vehicles will take time.

To address visibility challenges for drivers through the windshield, an affordable front-view camera display system would be beneficial. This system enables the application of image enhancement techniques tailored to different atmospheric and lighting conditions^24^. Specialized dehazing algorithms are used to improve images affected by fog, dust, or rain^25^. Low-light image enhancement techniques are beneficial for boosting visibility during early mornings or late evenings when lighting is poor. Nighttime visibility is further enhanced through techniques that brighten low-light areas while avoiding overexposure from the high beam glare of the oncoming vehicles. Choosing the most suitable algorithms for each specific adverse condition is crucial for optimal performance and enhancing the driving experience during night travel. Integrating these technologies with advanced image enhancement strategies creates a safer and more secure driving environment.

Real-time classification of road environments has emerged as a key component in intelligent transportation systems. The process often begins by leveraging deep learning models such as Convolutional Neural Networks (CNNs) to categorise driving scenes into conditions like day, night, rain, and fog^26^. Integrating such contextual classification early in the processing pipeline enhances the adaptability of subsequent tasks, allowing systems to dynamically respond to changing environments. As classification provides an understanding of the driving scenario, the next stage often involves enhancing image quality to improve visibility. In low-light conditions, techniques such as histogram equalization and CLAHE (Contrast Limited Adaptive Histogram Equalization) are applied to reveal road features like lane markings^27^. While effective in some cases, these methods tend to over-amplify noise, reducing image clarity. To balance contrast and preserve detail, the process evolves to include gamma correction techniques coupled with denoising filters such as non-local means or bilateral filtering. For more complex enhancement tasks, hybrid strategies combining multi-scale Retinex and dynamic gamma correction are introduced. Although these offer significant improvements in image quality, their computational demands pose challenges for real-time deployment on embedded systems.

Segmentation divides an image into meaningful regions, simplifying the extraction of specific objects or areas of interest^28^, while mosaicking seamlessly blends multiple images to create a unified and extensive view of a larger scene^29^. Edge detection identifies and enhances the boundaries of objects within an image, aiding in object separation and analysis^30^. Serial feature extraction involves step-by-step extraction of relevant features, forming a comprehensive representation of the visual data^31^. Pattern recognition identifies and categorizes specific patterns or objects within an image, relying on predefined models or templates. The effectiveness of image classification largely hinges on feature extraction, a specialized technique that reduces dimensionality in areas like image processing and pattern recognition, directly impacting classifier performance^32^. Convolutional Neural Networks (CNNs) excel in extracting high-level features from images, essential for distinguishing between different classes^33^.

The proposed research introduces an autonomous system designed to enhance road visibility as a secondary vision tool. The proposed system can also be regarded as an application of Frugal Engineering, as it emphasizes cost-effectiveness, simplicity, and resource optimization without compromising functionality. Such solutions are particularly relevant for low- and middle-income countries, where road safety remains a major concern, and affordable technologies can have a significant societal impact^34^. The road visibility will be hampered by changing environmental conditions which are identified as ‘road scenarios’ in this study. The system starts with ML-based road scenario classification to choose suitable image enhancement algorithms for each road scenario. These algorithms employ appropriate enhancement techniques to improve image quality such as foggy, rainy and nighttime, particularly against intense high-beam illumination at night. The process includes high-intensity image mapping to reduce glare in specific road areas, further enhanced by gamma correction for improved visual clarity. This methodical approach significantly reduces glare and improves road visibility, ensuring safer nighttime navigation. Implemented on a standalone Raspberry Pi 5 system, the dashboard display provides a clear view of the road in adverse conditions, thereby enhancing overall safety when the windshield becomes ineffective.

**Fig. 2:**
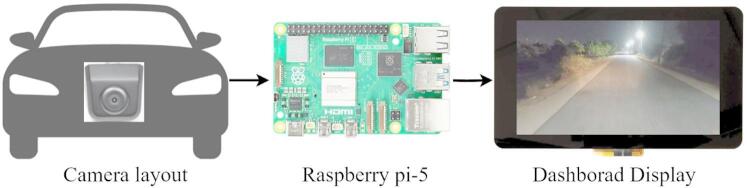
Hardware setup for proposed work.

## Methodology

**Fig. 3:**
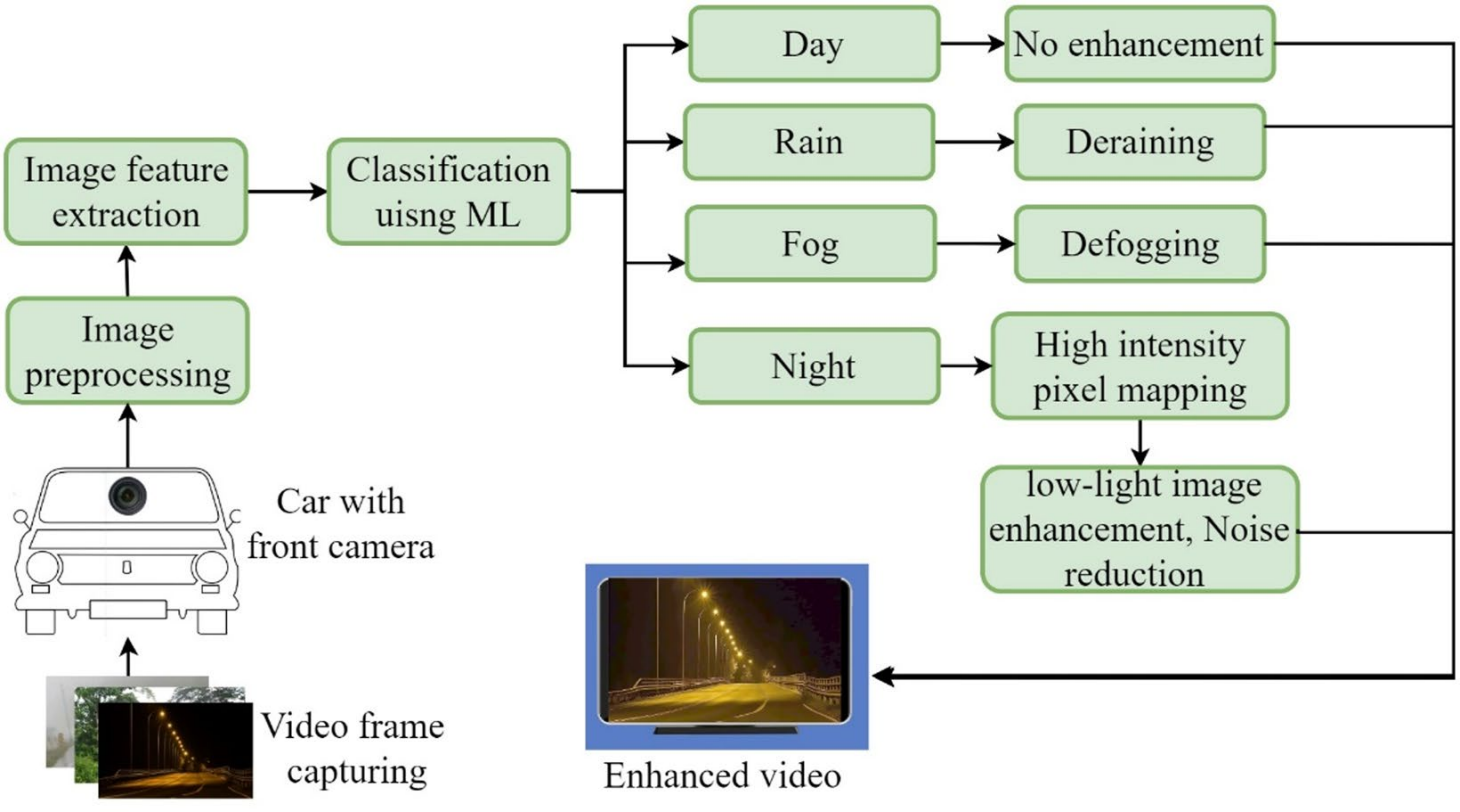
Proposed methodology.

The primary goal of this study is to ensure clear visibility for automobile drivers through a dashboard display when vision through the windshield is obscured under adverse conditions. Figure 2 illustrates the components and processes involved in the hardware model of the proposed work. A front-mounted camera captures real-time video frames at 30 frames per second, which are processed by a Raspberry Pi 5 and displayed on a 7-inch dashboard screen. In our prototype, the camera is mounted above the front bumper to ensure an unobstructed forward view of the road while minimizing reflections or distortions that may occur when placed behind the windshield. The system evaluates the current conditions and applies the relevant enhancement algorithms. Enhanced frames are then displayed at the same frame rate. The study is organized into three main sections: image classification with pretrained ML models, image enhancement, and practical implementation and testing. The training and development of models are performed on a computer equipped with an Intel i7 processor, 64 GB RAM, a 6 GB graphics card, and a 512 GB SSD. Figure 3 portrays the complete overview of the proposed methodology.

The proposed system is designed to automatically detect road scenarios and enhance visibility during adverse conditions through real-time video enhancement. The classification module achieved a frame rate of 9 fps (frames per second), while the image enhancement modules performed at 28 fps for low-light enhancement, 25 fps for defogging, and 29 fps for deraining. It utilizes image features derived from pixel intensity variation, color channel information, contrast, and sharpness to improve classification accuracy. Integrating microcomputers in vehicles to support ADAS systems reflects the trend towards “computers on wheels,” and the use of a Raspberry Pi-based model makes this system affordable and cost-effective within the ADAS field.

## Image classification with ML models

Machine learning techniques classify images by leveraging algorithms that learn from a dataset of labelled images. Custom features, such as brightness, and contrast, extracted from images and organized in a CSV file, are used during the training phase. Once trained, the model classifies new images by comparing their features to the learned patterns. The effectiveness of this classification depends on the quality of the training data and the ability of the model to generalize.

### Data collection

Images were captured from the front mounted camera, including daytime, nighttime, foggy, and rainy conditions, used for creating a diverse dataset. The images were acquired from two-way urban and rural roads, capturing diverse driving conditions. During video recording, vehicle speeds ranged between 30 and 50 km/h. The roads experienced varying traffic densities, with urban areas generally having higher traffic and rural roads encountering lower traffic levels depending on the location. This variation ensures a comprehensive dataset covering different real-world driving scenarios. For each scenario, 200 video frames were selected for classification. To enhance the dataset for machine learning, five augmentation techniques were applied including, zoom, flipping, blurring, rotation and noise addition, resulting in a total of 1,200 images per class. This process provided a dataset of 4,800 images for classification. Table 1 illustrates the augmentation technique in detail. Augmentation also aids in real-world implementation by addressing potential variations in camera angles, zoom capabilities, and image perspectives due to vehicle motion. Temporary blurring from focusing issues or lens contaminants, as well as differences in camera placement and driving side, can impact image quality. Training models on horizontally flipped images can help mitigate these variations, and adding noise ensures consistent performance across cameras of varying qualities.

**Table 1:** Details of augmentation techniques.

Augmentation	Value
Zooming	+ 25%
Blurring	Gaussian
Addition of noise	Random
Flipping- horizontal	90°
Rotation - both clockwise, anticlockwise	0° − 20°

### Impact of features

Basic images features which are suitable for this classification is used in this work. The features used here are brightness, contrast, sharpness, entropy, skewness, kurtosis, edge density, blue channel mean, red channel mean, green channel mean, hue channel mean, saturation channel mean, value channel mean, blue channel standard deviation, red channel standard deviation, and green channel standard deviation.

#### Brightness

Brightness measures the average pixel value of an image, indicating its overall light intensity. Daytime images generally exhibit high brightness due to natural sunlight. In contrast, nighttime images have significantly lower brightness due to the lack of sunlight and limited artificial lighting. Foggy images may also have reduced brightness compared to clear daytime images, as light scattering by fog particles diffuses the light. Similarly, rainy images can display lower brightness, particularly when dense rain clouds block sunlight.

#### Contrast

Contrast represents the difference in intensity between the darkest and lightest areas of an image. High contrast signifies sharp differences, which is typical in sunny daytime images with strong shadows. Nighttime images can also have high contrast due to bright artificial lights against a dark background. Foggy images usually show low contrast because the fog scatters light, giving the scene a hazy and muted appearance. Rainy images might exhibit moderate contrast, depending on the intensity of the rain and the extent of cloud cover.

#### Sharpness

Sharpness, measured by the variance of the Laplacian, indicates the clarity of edges in an image. Clear daytime images exhibit high sharpness with well-defined edges. Nighttime images can also be sharp if illuminated by artificial lights, but they may have areas with very low sharpness due to darkness. Foggy images typically have low sharpness since the fog diffuses light, causing edges to blur. Rainy images can have varying sharpness; light rain might cause slight blurring, while heavy rain can lead to significant blurring.

#### Edge density

Edge density, derived from the Canny edge detector, measures the number of edges in an image. Daytime images with clear visibility typically have high edge density due to well-defined objects and structures. Nighttime images can have moderate to high edge density if illuminated, but unlit areas will exhibit low edge density. Foggy images generally have low edge density as the fog blurs edges, making objects less distinct. Rainy images may show reduced edge density, especially if the rain causes motion blur or obscures objects.

#### Colour distribution (RGB means)

Colour distribution examines the average values of the red, green, and blue channels. Daytime images generally feature balanced and vibrant colour distributions. Nighttime images often display a higher blue channel due to bluish artificial lighting. Foggy images have muted colour distributions, frequently appearing greyish due to light scattering by fog particles. Rainy images tend to show less vibrant colours and more muted tones because of the overcast sky and reduced visibility.

#### Colour standard deviation (RGB colour space)

Colour standard deviation measures the variation within each colour channel. High variation during the daytime indicates a diverse and dynamic scene with a range of colours and lighting conditions. Nighttime images can show high variation in areas with artificial lighting but low variation in darker areas. Foggy images generally exhibit low colour variation due to the uniform scattering of light. Rainy images may have moderate colour variation, depending on the density of rain and cloud cover.

#### Entropy

Entropy measures the randomness or complexity of the pixel intensity distribution. Daytime images typically have high entropy due to diverse scenes and lighting conditions. Nighttime images may have lower entropy in dark areas but higher entropy in illuminated regions. Foggy images usually exhibit lower entropy because of the uniformity caused by the fog. Rainy images can display varying levels of entropy; light rain might not significantly change the complexity, but heavy rain can reduce entropy by creating more uniform areas.

#### Skewness

Skewness indicates the asymmetry of the pixel intensity distribution. Daytime images typically have a distribution skewed towards higher intensity values due to abundant natural light. Nighttime images are skewed towards lower intensity values because of the overall darkness. Foggy images might have a near-normal distribution with less skew, as the fog evens out the light distribution. Rainy images can vary in skewness depending on the lighting conditions and the intensity of the rain.

#### Kurtosis

Kurtosis measures the sharpness of the peak in the pixel intensity distribution. Daytime images, with their varied and dynamic lighting, might exhibit moderate kurtosis. Nighttime images, with high contrast between lit and dark areas, can show high kurtosis due to sharp intensity peaks. Foggy images typically have low kurtosis as the fog creates a more uniform intensity distribution. Rainy images may display moderate to high kurtosis, influenced by the varying intensity caused by raindrops and cloud cover.

#### HSV means

Hue mean, saturation mean, and value mean offer an alternative analysis using the HSV colour space. Daytime images generally exhibit high saturation and value, indicating vivid colours and bright light. Nighttime images may have lower saturation but can still display high value in illuminated areas. Foggy images often show low saturation and value, reflecting muted and dull colours. Rainy images usually have reduced saturation and value compared to clear days, due to subdued lighting conditions and an overcast sky.

### Feature selection

A total of relevant features, including brightness, contrast, sharpness, entropy, skewness, kurtosis, edge density, RGB and HSV channel means and standard deviations, were extracted from 4,800 images and stored in a CSV file. These features were chosen because they represent lighting, sharpness, and visibility variations across day, night, fog, and rain conditions. The J48 decision tree algorithm, an implementation of the C4.5 algorithm, was employed for feature selection. J48 constructs a decision tree by recursively splitting the dataset based on features that provide the maximum information gain^35^. Feature selection aims to boost prediction accuracy by identifying key features and removing irrelevant ones. This process simplifies the model. Decision trees are often used for this purpose due to their clear and structured representation. They visually present classification rules with roots, branches, nodes, and leaves. In these trees, leaves represent class labels. Branches illustrate paths from the root to the leaves through nodes that contain classification features. Decision nodes are helpful to determine the most important feature based on a relevant criterion. During this process, features that are highly discriminative appear near the root nodes of the tree, while less relevant or redundant features are placed deeper or pruned. This property makes J48 effective not only for classification but also for identifying the most significant features. The J48 decision tree method is particularly effective for feature selection. In classifying images under different lighting conditions, numerous features are extracted and irrelevant features are discarded during the classification process^36^.

**Fig. 4:**
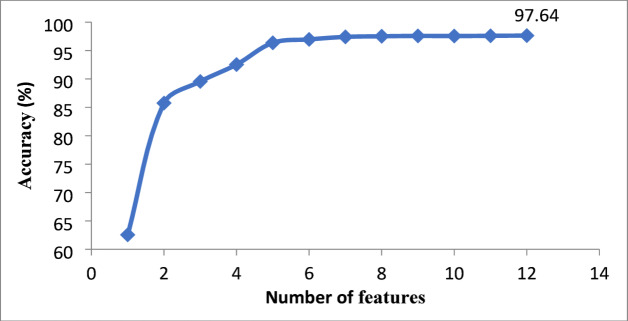
Variation of accuracy based on the number of features.

Of 16 features, 12 features have been used for making tree, namely green mean, edge density, sharpness, blue mean, saturation mean, red standard deviation, hue mean, skewness, kurtosis, contrast, entropy and, brightness. These features are used for road scenario classification in this priority order. The data showcases the incremental improvement in classification accuracy as additional features are incorporated into the model. Figure 4 depicts the effect of the number of features on classification accuracy. Initially, the model achieves an accuracy of 62.56% with only the most important feature, the green mean. The accuracy dramatically increases to 85.75% with the addition of edge density, highlighting the importance of combining features. As more features are included, the accuracy continues to rise, reaching 97.64% when all twelve features are used. This progression underscores the value of each feature in enhancing the ability of the model to differentiate between various image conditions such as normal daytime, foggy time, nighttime, and rainy time. The significant jumps in accuracy with the addition of early features, followed by gradual improvements, indicate that while key features provide substantial initial insights, a comprehensive feature set is crucial for achieving optimal classification performance.

### Classification

The total data set of 4800 images was divided into 80% and 20% for training and testing. A tenfold cross-validation is used for validation. Weka software is used as a platform for training and testing. Different ML algorithms have been used for classification including tree-based, lazy-based, Bayes-based, Meta classifiers and functional classifiers algorithms^37^.

## Image enhancement

Driving at night, especially on two-way roads, poses several challenges due to windshield transparency issues and high beam glare from opposite vehicles. The lack of streetlights and lane markings, along with potholes, irregular road surfaces, and unmarked speed breakers, further complicate night driving. Rainy conditions also reduce visibility and create slippery roads. Heavy rain obscures road signs, vehicles, and other obstacles. Raindrops on the windshield and glare from oncoming headlights worsen visibility. Water on the road increases the risk of skidding or hydroplaning, and puddles can hide hazards. Similarly, foggy conditions severely reduce visibility. This makes it difficult for drivers to judge distances accurately and react quickly. Headlights reflecting off the fog create a bright, white wall, further diminishing visibility. Moisture in the fog can create slippery surfaces. Implementing a display system with image enhancement algorithms can improve driver safety and convenience.

### Nighttime - low light image enhancement

Nighttime road images present two contrasting elements. The first is high-intensity light sources, such as high-beam lights, which make image interpretation difficult. The second element is the darker regions, like the sides of the road. A high-intensity pixel mapping technique is used to reduce the intensity of high-beam light pixels. This is followed by gamma correction to enhance low-intensity regions, and finally, noise reduction is applied to the enhanced image^27^.

If a pixel intensity I is above the threshold T,

1 $$\:I^{\prime}=\:I\times\:factor$$

where I′ is the corrected intensity and the factor is a value less than 1 (e.g., 0.8). For pixels where I ≤ T, the intensity remains unchanged.

Gamma correction is applied after high-intensity pixel reduction and helps to enhance low-light regions. It adjusts brightness and contrast to make the image more perceptually uniform and to improve its overall appearance according to human visual perception. A precomputed lookup table can be used to efficiently map the original pixel values to their gamma-corrected counterparts. Gamma correction is advantageous due to its relatively low computational cost.

The gamma correction equation is,

2 $$\:I^{\prime}\:=\:{I}^{\gamma\:}$$

where I is the original pixel intensity, I′ is the gamma-corrected intensity, and γ is the gamma value. For γ > 1, the image becomes darker, and for γ < 1, the image becomes brighter^38^.

Further, a Fast Non-Local Means Denoising technique can be used for noise reduction. The Non-Local Means algorithm functions by comparing a patch, which is a small window of pixels, centred around each pixel with other patches in the image. Unlike simpler methods like Gaussian or median filtering, it doesn’t just average neighbouring pixels. Instead, it averages all pixels in the image with similar patches, no matter how far apart. A Gaussian-weighted Euclidean distance determines the similarity between these patches^39^.

### Foggy atmosphere

Using the Dark Channel Prior method, the defogging technique aims to enhance visibility in images obscured by fog. The process begins by estimating the fog’s effect through the transmission map. This map t(x) is computed as,

3 $$\:t\left(x\right)\:=\:1-\omega\:.{J}_{d}\left(\:\frac{I\left(x\right)}{A}\:\right)$$

where J_d_ is the dark channel of the normalized image I(x)/A, and ω is a parameter controlling the contrast. The dark channel J_d_ ​ is calculated as the minimum value of the image’s RGB channels in a local patch:

4 $$\:{J}_{d}\left(x\right)\:=\:{min}_{y\in\:\varOmega\:\left(x\right)}\:\left({min}_{c\in\:\{R,G,B\}}{I}_{c}\right(y\left)\right)$$

Next, the image is corrected to remove the fog using the estimated transmission map. This involves adjusting the original image I(x) to recover the scene,

5 $$\:{I}_{recovered}\left(x\right)=\frac{\:I\left(x\right)-A}{\:t\left(x\right)}+A$$

where A is the atmospheric light. This equation effectively brightens the image where fog is denser, resulting in a clearer, defogged image^40^.

### Rainy atmosphere

It starts by capturing video frames and converting each frame to grayscale for processing. The guided filter, an edge-preserving smoothing filter, is applied to the grayscale image to separate the rain streaks from the underlying scene. The guided filter involves calculating local means and variances of the image and using these to compute coefficients for linear filtering. Specifically, for an input image I and a guidance image p, the mean and variance are computed within a window of radius r to derive coefficients a and b such that q = a ⋅ I + b, where q is the smoothed output^41^. The algorithm calculates a and b as follows:

6 $$\:{mean}_{I}=\:boxfilter\left(I\right)$$

7 $$\:{mean}_{p}=boxfilter\left(p\right)$$

8 $$\:{mean}_{Ip}\:=\:boxfilter(I.p)$$

9 $$\:{cov}_{Ip}={mean}_{p}-\:{mean}_{I}\:.{mean}_{p}$$

10 $$\:{var}_{I}=boxfilter\left({I}^{2}\right)-{\:{mean}_{I}}^{2}$$

11 $$\:a\:=\:\frac{{cov}_{Ip}}{{var}_{I}+\epsilon\:}$$

12 $$\:b\:=\:{mean}_{p}-\:{a.mean}_{I}$$

$\:{cov}_{Ip}\:$is the covariance between the guidance image I and the input image p.

​$\:{var}_{I}\:\:$is the variance of the guidance image I.

ε is a small regularisation term to avoid division by zero.

$\:{mean}_{I}$ is the mean of the input image I.

$\:{mean}_{p}$ is the mean of the guidance image p.

## Results and discussion

### ML classification

To evaluate optimal performance, this study compares the best-performing machine learning models from the following categories; decision tree-based models, Bayesian models, lazy classifiers, meta-classifiers, and function-based models. Table 2 lists the top-performing algorithms, along with their training, testing, validation accuracies, and time required for model building. In Table 2, Bayes classifiers contribute a probabilistic approach, leveraging feature relationships for quick and efficient classification. In this study, BayesNet and Naive Bayes techniques are used and their classification results are shown in Table 2. Functional classifier models like Logistic, Multilayer Perceptron and Simple Logistic classifiers capture intricate patterns in the features extracted from images. Lazy classifiers like K-Nearest Neighbors (KNN) and K-Star, are known to effectively handle complex decision boundaries. Meta classifiers like Classification via Regression and Random Committee, aggregate predictions from multiple base classifiers. Classification results obtained from the above mentioned Functional, Meta and Lazy classifiers are listed in Table 2 as well. Tree-based models, such as J48, Random Forest, Optimized Forest, and nine others were also used in this study, owing to their robustness in managing large feature sets and interpretable results. This comprehensive selection of classifiers allowed for an optimal choice based on performance across varying road and weather conditions, ensuring the most accurate and reliable classification for each scenario^42^. The four ML classifiers, Random Forest, Multilayer Perceptron, Random Committee, and Optimized Forest performed superior to others by their testing accuracy.

#### Random forest

Random Forest is an ensemble learning technique that enhances classification accuracy and robustness by combining multiple decision trees. Each tree is trained on a different subset of the data and features. This introduces diversity among the trees and helps reduce overfitting while improving generalization. The final classification is determined by aggregating the results from all the decision trees. This is typically done through majority voting or averaging. By leveraging the strengths of each tree, Random Forest produces more reliable and accurate predictions. The method excels with complex datasets and a broad range of features. It adapts well to different data patterns and performs strongly even in noisy conditions.

#### Multilayer perceptron (MLP)

MLP is a type of feedforward neural network. It features fully connected neurons and uses nonlinear activation functions. This structure makes it especially effective for distinguishing data that is not linearly separable. The MLP includes an input layer, which processes raw features from road images or data. It also has one or more hidden layers that detect complex patterns through nonlinear transformations. The output layer then produces the final classification, such as “daytime,” “foggy,” “nighttime,” or “rainy.” Training involves backpropagation and gradient descent, which adjust the weights of the network to minimise prediction errors. MLP is highly effective for classifying road scenarios due to its ability to manage a variety of features, including brightness, texture, and edge information. Its flexibility allows it to adapt to different lighting and weather conditions. This adaptability makes it well-suited for identifying complex and dynamic road scenarios. The capability of the network to learn intricate patterns and relationships ensures accurate classifications, even when dealing with data that is not linearly separable.

**Table 2:** Training, testing, and validation accuracy of different ML classifiers.

S.no	ML	Training accuracy	Time (s)	Testing accuracy	Time (s)	Validation accuracy	Time (s)
1	BayesNet	95.02	0.07	93.64	0.03	94.29	0.05
2	Naive Bayes	90.57	0.01	90.83	0.00	90.26	0.00
3	Logistic	97.94	0.35	97.08	0.30	97.57	0.28
**4**	**Multilayer perceptron**	**99.27**	**1.82**	**98.22**	**1.79**	**98.82**	**1.86**
5	Simple logistic	97.81	0.41	97.91	0.30	97.76	0.29
6	KNN	100.00	0.00	97.91	0.00	98.72	0.00
7	K-star	100.00	0.00	97.60	0.00	98.80	0.00
8	Classification via regression	99.32	0.12	97.50	0.12	98.02	0.12
**9**	**Random Committee**	**100.00**	**0.13**	**98.43**	**0.05**	**98.43**	**0.07**
10	Best First tree	99.14	0.18	96.87	0.30	96.71	0.11
11	Cs forest	99.19	2.45	97.18	2.23	97.31	2.22
12	Extra tree	100	0.01	94.68	0.01	95.83	0.00
13	J48	99.34	0.02	96.87	0.02	96.82	0.02
14	J48 consolidated	99.21	0.08	96.45	0.07	96.82	0.08
15	J48graft	99.34	0.05	96.66	0.04	96.92	0.04
16	Logistic Model Tree	99.81	1.41	97.91	1.30	98.54	1.30
17	Naïve Bayes Tree	99.76	1.95	97.29	1.83	96.74	1.83
**18**	**Optimized Forest**	**100.00**	**11.38**	**98.33**	**1.66**	**98.51**	**11.36**
**19**	**Random Forest**	**100.00**	**0.45**	**98.22**	**0.46**	**98.54**	**0.45**
20	Random Tree	100.00	0.01	97.08	0.01	96.61	0.01
21	Simple Cart	99.11	0.35	96.66	0.12	97.03	0.12

#### Random committee

Random Committee is an ensemble learning method that improves classification accuracy and robustness by combining multiple classifiers. It involves training several individual classifiers, often decision trees, on different subsets of data or features. This introduces diversity among the classifiers, helping to reduce overfitting and enhance generalization. The final prediction is made by aggregating the outputs from all classifiers, typically using voting or averaging methods. This approach is particularly effective for handling complex classification tasks. It can manage a broad range of features and adapt to various data patterns. By leveraging the strengths of different models and addressing their weaknesses, the Random Committee achieves higher accuracy and robustness. This makes it well-suited for scenarios involving high-dimensional data or noisy environments.

#### Optimized forest

Optimized Forest is an advanced ensemble learning method that builds on traditional Random Forests with various optimizations. It constructs multiple decision trees, each trained on different subsets of data and features, similar to Random Forest. However, Optimized Forest introduces additional strategies to enhance performance. These strategies include feature selection, hyperparameter tuning, and advanced tree-building algorithms. The final prediction is made by aggregating the results from all optimized trees, usually through majority voting or averaging. Optimized Forest excels in handling complex datasets and adapting to different data patterns. By refining various aspects of the forest, it achieves higher accuracy and robustness compared to traditional Random Forests. This makes it particularly effective for scenarios involving high-dimensional data or noisy inputs.

Among the classifiers tested, Random Committee achieved the highest classification accuracy of 98.43%, followed by Optimised Forest (98.33%), Random Forest (98.22%), and Multilayer Perceptron (MLP) (98.22%). These algorithms demonstrated strong generalisation and stability across varying lighting and weather conditions. The Random Committee classifier, an ensemble of multiple base learners, effectively reduces overfitting and variance. Optimised Forest enhances Random Forest by tuning hyperparameters for better accuracy. MLP, a neural network capable of modelling nonlinear relationships, was particularly effective in handling complex image features. The consistent performance of these classifiers under diverse road scenarios justifies their selection for detailed evaluation in this study.

**Fig. 5:**
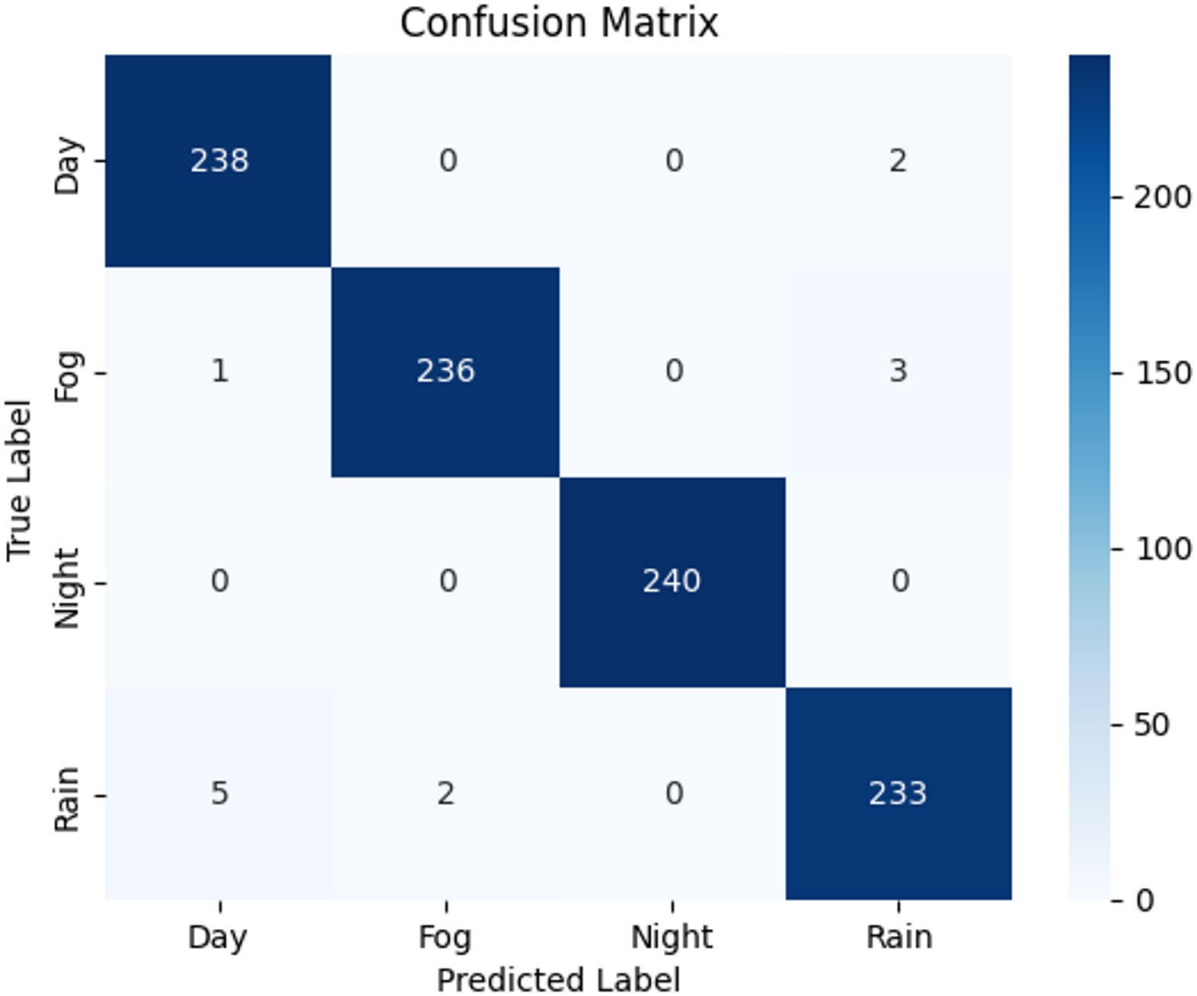
Confusion matrix of the test set using random committee classifier.

**Table 3:** Performance metrics for classification Evaluation.

Class	Precision	Recall	F-measure
Day	0.975	0.992	0.983
Fog	0.992	0.983	0.987
Night	1	1	1
Rain	0.979	0.971	0.975
Average	0.986	0.986	0.986

Hyperparameter tuning is crucial in the machine learning process to achieve a balance between model accuracy and computational efficiency, preventing underfitting and overfitting. By adjusting parameters such as the learning rate or number of trees, tuning enables models to capture complex patterns accurately while maintaining responsiveness. Without tuning, models risk reduced performance and reliability in real-world scenarios, making it essential for achieving accurate, adaptable, and efficient results across diverse conditions. Among the ML models used, the Random Committee classifier demonstrated the best performance (Table 2), After hyperparameter tuning (Batch size-100, Number of iterations-30, seed-1) the model achieved a training accuracy of 100%, testing accuracy of 98.64 and a validation accuracy of 98.62% with minimal model-building time. Figure 5 shows the confusion matrix for the test dataset, highlighting high classification accuracy, especially for Day and Night classes, which had minimal misclassification. Rain had a slightly higher misclassification rate, occasionally being confused with Day and Fog, likely due to overlapping features. These results identified the Random Committee model as the top-performing classifier in this study. Different evaluation metric such as precision measure and F-measure are included in Table 3. The trained model was saved in Python packet (.pkt) format and deployed on a Raspberry Pi, where this format is optimized for resource-constrained devices, making it ideal for edge processing.

In evaluating the classifier performance through confusion matrices, specific failure modes were observed under challenging conditions such as extreme weather and lighting. For instance, Rain images were often misclassified as Day or Fog across multiple classifiers, including Naive Bayes and BayesNet. This misclassification can be attributed to overlapping visual features, such as low contrast and blurriness, present in both fog and rain scenes. Additionally, Extra Tree showed occasional confusion between “Day” and “Night” images, likely caused by low-light daytime scenarios or high-intensity glare resembling nighttime lighting. Naive Bayes, in particular, demonstrated limitations in handling feature dependencies, leading to higher misclassification rates in fog and rain scenes where complex feature interactions occur.

The trained model was validated across four distinct classes using two publicly available Kaggle datasets: the ‘night-to-day’ dataset (for day and night) and the ‘weather image recognition’ dataset (for fog and rain). A sample of 100 images from each dataset was tested with the proposed model, achieving a classification accuracy of 80%, which underscores the versatility of the model in handling diverse geographical conditions.

### Comparison with contemporary methods

Table 4. indicates different methods used for the classification of similar works by other authors. From the literature survey, it is noted that ResNet-101, a customised CNN and DNN have been used in road scene classification resulting in varying degrees of accuracy. The proposed study gives the best classification accuracy of 98.64% among others.

**Table 4:** Comparison with contemporary methods.

Experiment name	Network used	Classification accuracy (%)
Road scene classification^43^	ResNet-101	88.00
Road surface classification (RTK dataset)^44^	Custom CNN network	94.57
Road type classification^45^	Custom deep neural network	87.38
Road visibility condition classification (proposed model)	Random committee with customised features	**98.64**

### Image enhancement

The selection of image enhancement algorithms was guided by both their effectiveness in specific adverse weather conditions and their suitability for real-time performance on limited hardware. For nighttime conditions, gamma correction was used to brighten low-intensity regions after reducing high-beam glare using a high-intensity pixel mapping technique.

This combination was found to be both efficient and visually effective under low-light scenarios. Fast Non-Local Means denoising was used to preserve edges while reducing noise, particularly effective in enhancing nighttime images without introducing blurring. For foggy conditions, the Dark Channel Prior (DCP) method was employed due to its strong performance in restoring contrast and visibility in hazy environments. While computationally more intensive than basic histogram equalization, DCP was feasible under proposed hardware setup and yielded better clarity. In rainy scenarios, the guided filter was selected because of its ability to separate rain streaks from background details efficiently, making it suitable for real-time applications. Although certain methods such as deep learning-based enhancement may offer higher quality results, the chosen techniques offer a balanced trade-off between enhancement quality and computational feasibility.

**Fig. 6:**
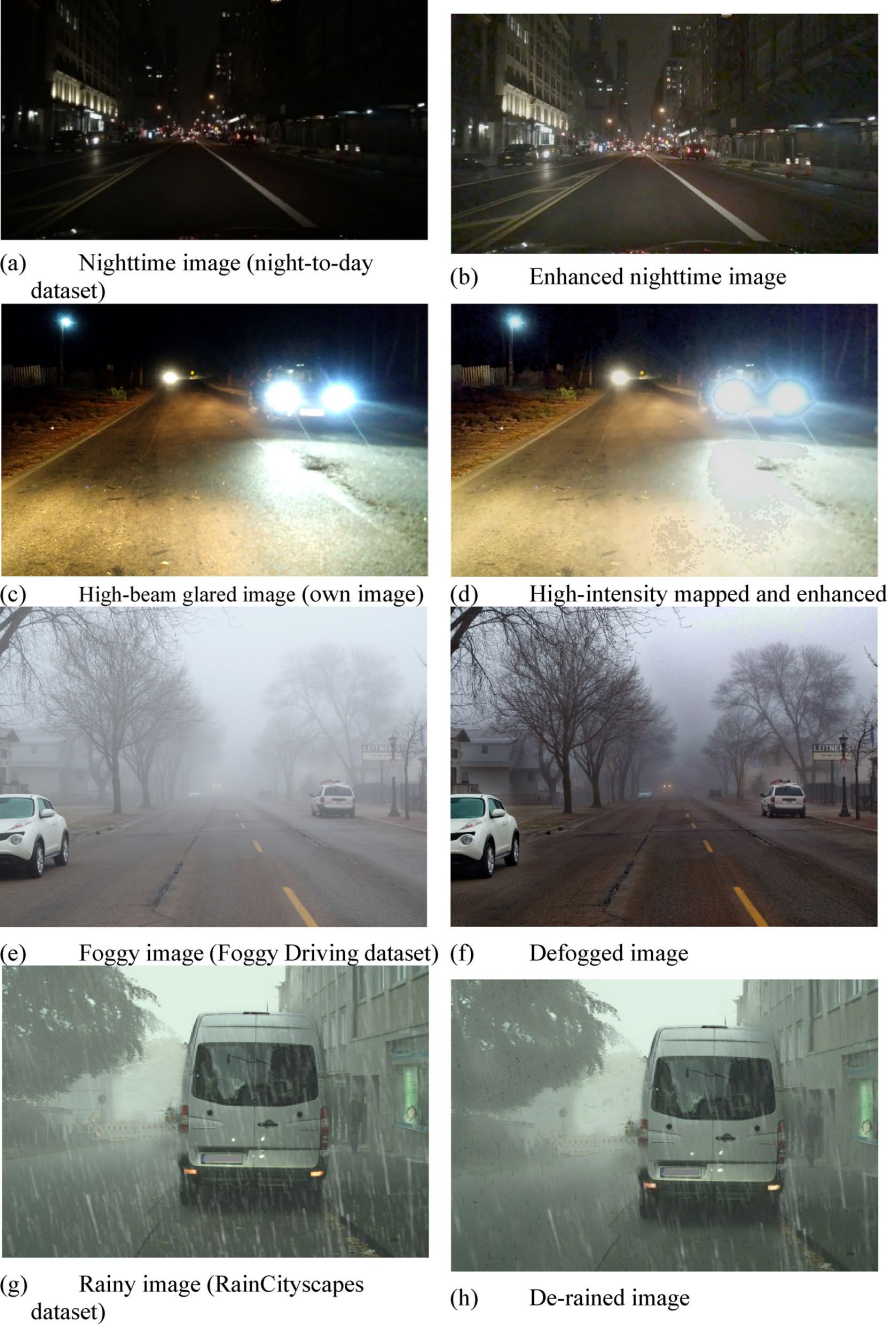
Enhancement results of images captured under various adverse conditions.

Figure 6 shows the original and enhanced versions of images captured under different adverse weather conditions. Due to hardware constraints, the enhancement techniques have been simplified, with only less complex methods employed. Figures 6(a)^46^, (c), (e)^47^, and (g)^48^show the original nighttime, glared, foggy and rainy images respectively. Noise reduction and low-light image enhancement, which are crucial for nighttime conditions, are illustrated in Fig. 6(b). When a vehicle with high-beam headlights approaches, glare can obscure the entire windshield, making it difficult for the driver to see ahead. However, using algorithms that reduce high-beam intensity, combined with gamma correction and noise cancellation, improves visibility as shown in Fig. 6(d). Defogging is demonstrated in Fig. 6(f), and de-raining is depicted in Fig. 6(h). Although the de-raining technique did not perform optimally, it provided rapid enhancement results. In very dense situations, the proposed system cannot provide enhanced results due to the lack of details in the images however potential improvements can be possible with advanced sensor fusions.

### Integration with advanced driver assistance systems (ADAS)

The proposed road scenario classification and enhancement system can be used to enhance current ADAS functions. The system integrates as an additional vision module, providing real-time, enhanced visual data to the ADAS. It is especially useful in adverse conditions like rain, fog, and low light, where traditional cameras often struggle. By supplying improved images to the ADAS pipeline, the system boosts perception accuracy. This ensures that critical tasks such as lane detection, pedestrian recognition, and adaptive cruise control continue to work effectively, even with compromised windshield visibility.

**Fig. 7:**
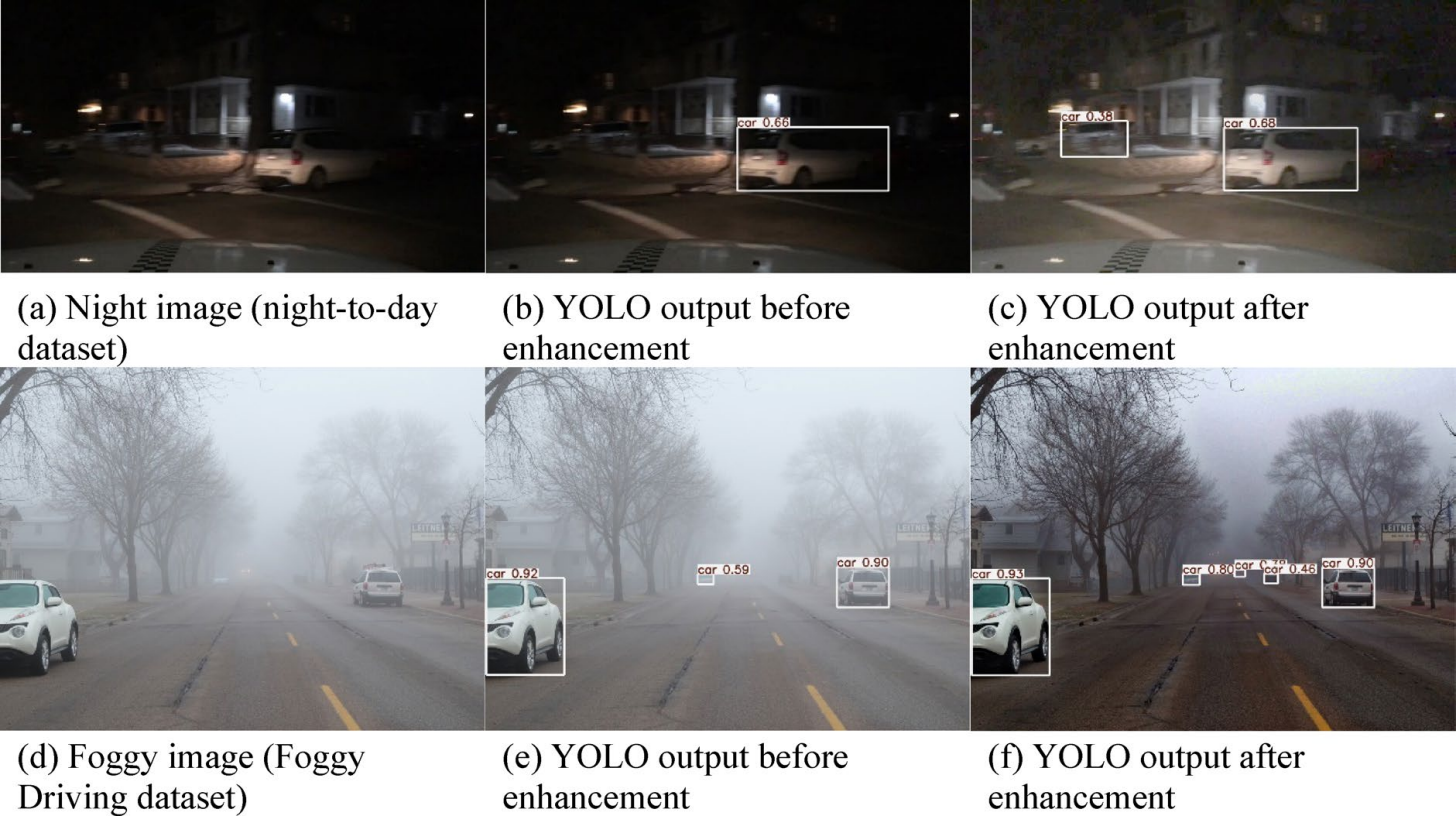
YOLO object detection using Traffic Agent Classification algorithm: (**a**) Night image; (**b**) YOLOv5 detected night image; (**c**) YOLOv5 detected enhanced night image; (**d**) Foggy image; (**e**) YOLOv5 detected foggy image; (**f**) YOLOv5 detected enhanced foggy image.

Figure 7 compares the performance of the YOLOv5 Traffic Agent Classifier using both original and enhanced images. The proposed model demonstrates significant improvements in object detection subsequent to image enhancement^49^. Although machine vision and human perception differ, the image enhancement model originally designed to enhance visual quality, has proven effective in improving object detection as well. This experiment examines a selection of images enhanced using custom techniques. Both original and enhanced images were processed through the YOLOv5 detection system, with enhanced images often yielding better results. For example, in Fig. 7(c), the low light-enhanced image detects two cars, whereas, in Fig. 7(b), the YOLOv5 detects only one car due to the darkness and slight blur of the original image. Similarly, under foggy atmosphere shown in Fig. 7(d), the YOLOv5 performance improved while processing the enhanced image rather than processing the original image. Figure 7(e) shows that YOLOv5 detected three cars from the original foggy image. However, Fig. 7(f) shows that five cars are detected from the enhanced foggy image. These results demonstrate the improved performance of the proposed algorithm. The broad applicability of proposed technique is underscored in this analysis.

## Hardware implementation

**Fig. 8:**
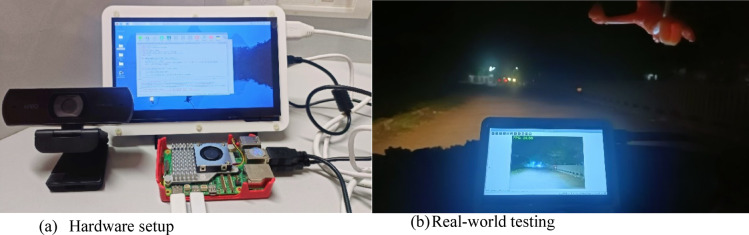
(**a**) Hardware components for the proposed system, (**b**) The testing of the proposed system on nighttime.

A Raspberry Pi 5, a webcam, and a 7-inch display are used as the hardware for this implementation. The webcam used for testing has specifications such as FPS – 60 @ 1080p, 78-degree field of view, 2 megapixels and a USB interface. The webcam is mounted at the front of the vehicle to capture clear road images, and the display is connected to the dashboard. Figure 8(a) shows the hardware with connections^50^.

The proposed system performs scenario classification every minute to identify current road conditions. While the Random Committee algorithm can achieve 9 frames per second (fps) on this hardware, the real-time video from the camera is enhanced and sent to the display as a secondary road view when the scene visibility through the windshield becomes poor in adverse conditions. In normal conditions, the video can be used for safety and surveillance. Table 5 shows the frame rates achieved by various algorithms with a 512 × 288 image size on Raspberry Pi 5. The system is designed to minimize driver distraction and cognitive load, featuring a simple and intuitive user interface that requires minimal interaction. Fully automated, it detects road conditions and enhances images without manual input. The dashboard display is aligned with the natural line of sight of the driver and activates only when visibility is compromised, ensuring it is used only when beneficial. Real-time processing provides immediate, clutter-free enhanced visuals, promoting safer driving in challenging conditions. The system was installed in a car with a USB camera on the windshield and a dashboard display powered by a temporary 5 V power bank for real-world testing. Figure 8(b) shows the test conducted at night time. Test results matched with indoor evaluation, demonstrating the potential of the system for effective, real-time use across various driving scenarios.

The operating range for the hardware is 0 °C to 60 °C for the Raspberry Pi 5 and USB webcam, and − 20 °C to 70 °C for the display according to manufacturer specifications. Prolonged exposure to temperatures outside these ranges could degrade hardware. So future upgrades will include weather-resistant enclosures to protect against moisture, dust, and extreme temperatures. Active cooling solutions, like heat sinks or fans, can prevent overheating in high temperatures. Vibration-damping mounts will also protect hardware from road-induced vibrations, enhancing durability and stability in harsh conditions.

This Raspberry Pi-based system focuses on delivering essential visibility improvements in challenging conditions like fog, rain, and low light at a fraction of the cost of other ADAS systems. In contrast, the ADAS solutions, such as Nissan ProPILOT Assist and Honda Sensing, come at a higher price, offering automation features like lane-keeping and adaptive cruise control that enhance convenience but do not emphasize visibility improvements. To enhance the performance of the proposed model hardware accelerators like Google Coral can be integrated. Additionally, model optimization techniques such as quantization and pruning can help reduce computational load. Lowering input resolution or frame rate can further improve real-time performance, enabling effective use of simpler algorithms while preserving cost-effectiveness. Future work will explore integration with speed-based activation, where the system will function optimally at lower speeds to prevent unnecessary driver distraction at high speeds. Additionally, alternative display and camera placements will be considered to ensure that the enhanced images are positioned in the most ergonomically appropriate locations while maintaining clear visibility for the driver. The use of deep learning models also be a part of future work.

For future scalability, the system could be enhanced using advanced platforms like the NVIDIA Jetson Nano or Xavier, which offer greater computational power for real-time processing of complex models. Integrating high-resolution cameras and sensors, such as LiDAR and radar, would improve environmental data collection, particularly in adverse weather conditions. Future integration with ADAS features, such as lane departure warnings, pedestrian detection, and traffic sign recognition, could extend its application to autonomous and semi-autonomous vehicles.

**Table 5:** Frames per second were obtained after applying enhancement techniques.

Enhancement technique	Frames per second
Low light image enhancement	28
De-fogging	25
De-raining	29

## Conclusion

This research proposes a practical solution to help drivers when the windscreen visibility is obscured by low light, high beam glare, fog, or rain. The system, developed with a Raspberry Pi 5, USB camera, and dashboard display, utilizes top-performing machine learning algorithms to assess road conditions. The Random Committee classifier achieves a maximum accuracy of 98.64% at 9 fps. After classification, tailored image enhancement techniques are applied to improve visibility. The current setup achieves over 25 fps with all image enhancement techniques at a resolution of 512 × 288, as implemented in this study. However, the limited computational capacity of the Raspberry Pi restricts the use of more complex algorithms, which could further enhance image clarity and response time in challenging scenarios. A more advanced platform, such as the NVIDIA Jetson series, could support deep learning algorithms, enhancing adaptability across diverse road and weather conditions. Improving the adaptability of the system through broader datasets and advanced image processing could make it a robust vision tool, with potential applications in future ADAS and autonomous vehicles.

## Data Availability

The datasets used and/or analysed during the current study are available from the corresponding author on reasonable request.
